# microRNA Heterogeneity, Innate-Immune Defense and the Efficacy of SARS-CoV-2 Infection—A Commentary

**DOI:** 10.3390/ncrna7020037

**Published:** 2021-06-18

**Authors:** Walter J. Lukiw

**Affiliations:** 1 LSU Neuroscience Center, Louisiana State University Health Science Center, New Orleans, LA 70112, USA; wlukiw@lsuhsc.edu; 2 Department of Ophthalmology, Louisiana State University Health Science Center, New Orleans, LA 70112, USA; 3 Department Neurology, Louisiana State University Health Science Center, New Orleans, LA 70112, USA

**Keywords:** COVID-19, *Homo sapien* (hsa), messenger RNA (mRNA), microRNA (miRNA), severe acute respiratory syndrome coronavirus-2 (SARS-CoV-2)

## Abstract

Severe acute respiratory syndrome coronavirus-2 (SARS-CoV-2), a member of the genus *Betacoronavirus* in the family *Coronaviridae*, possesses an unusually large single-stranded viral RNA (ssvRNA) genome of about ~29,811 nucleotides (nt) that causes severe and acute respiratory distress and a highly lethal viral pneumonia known as COVID-19. COVID-19 also presents with multiple ancillary systemic diseases and often involves cardiovascular, inflammatory, and/or neurological complications. Pathological viral genomes consisting of ssvRNA, like cellular messenger RNA (mRNA), are susceptible to attack, destruction, neutralization, and/or modulation by naturally occurring small non-coding RNAs (sncRNAs) within the host cell, some of which are known as microRNAs (miRNAs). This paper proposes that the actions of the 2650 known human miRNAs and other sncRNAs form the basis for an under-recognized and unappreciated innate-immune regulator of ssvRNA viral genome activities and have implications for the efficiency of SARS-CoV-2 invasion, infection, and replication. Recent research indicates that both miRNA and mRNA abundance, speciation, and complexity varies widely amongst human individuals, and this may: **(i)** In part explain the variability in the innate-immune immunological and pathophysiological response of different human individuals to the initiation and progression of SARS-CoV-2 infection in multiple tissue types; and **(ii)** further support our understanding of human biochemical and genetic individuality and the variable resistance of individuals to ssvRNA-mediated viral infection and disease. This commentary will briefly address current findings and concepts in this fascinating research area of non-coding RNA and innate-immunity with special reference to natural host miRNAs, SARS-CoV-2, and the current COVID-19 pandemic.

## 1. Introduction—Overview

miRNAs (microRNAs) are a family of short non-coding RNA (sncRNA) 18–23 nucleotides (nt) in length that regulate gene expression in human cells using a post-transcriptional regulatory mechanism [1]. Typically generated by RNA polymerase II (RNAPII)-based transcription from genomic DNA and pre-miRNA processing mechanisms, human cells typically contain about ~2650 different miRNA species, although miRNA populations vary widely amongst different cell types and amongst individuals of the same species (miRBase v.22; GENCODE data v.29) [2,3,4,5]. Different cell types contain a different complement of miRNAs that vary in abundance, speciation, and complexity during development, homeostatic day-to-day function, aging, and disease [4,5,6]. Although multiple mechanisms exist, the general mode of miRNA action is to bind to the 3′-untranslated region (3’-UTR) of their target messenger RNA (mRNA) via complementary base-pair interactions; in doing so, this miRNA-mRNA hybrid: **(i)** Can impair or block normal mRNA transit through the cleft of the eukaryotic 80S ribosome [4,5,7]; **(ii)** represses gene expression post-transcriptionally by destabilizing and down-regulating their target mRNA(s) [1,3,4]; and **(iii)** reduce protein production and repress genetic information encoded in that target mRNA [1,2,3,4,5]. The majority of miRNA–mRNA interactions, biogenesis, and function in any human cell remain largely unidentified, poorly characterized, and incompletely understood, although rapid research progress is being made. Multiple miRNA-mRNA interactions are known to be especially important in human neurological health and disease [2,5,6]. Interestingly, as single-stranded RNAs, messenger RNA (mRNA) and single-stranded viral RNA (ssvRNA) appear to be interchangeable targets for miRNA and/or sncRNA action [3,4,5,6,7,8]. That is, any single-stranded RNA or ssvRNA encountered by a host miRNA that is sufficiently complementary will be down-regulated, and its potential biological activity will be accordingly diminished. 

One of the more remarkable aspects of human genetic biology are the widely observed differences and variability between individuals in: **(i)** Their complement of expressed mRNA even among carefully age-, tissue- and gender-matched cell and tissue samples [5,8,9]; **(ii)** the highly variable composition and staggering genetic diversity of the nucleic acids contained within microbial populations which constitute the human gastrointestinal (GI)-tract microbiome (that contribute at least ~22 million genes to the human metagenome) [10]; and **(iii)** the repertoire of microRNAs and the diversity in the quantity and kind of miRNAs and mRNAs which exhibit a surprisingly wide variation among individuals [11,12,13]. Put another way, human individuals each have their own resident complement of miRNA, mRNA, and microbiome-derived nucleic acids that generate significant individual variation in ssRNA populations [8,9,10,11,12,13,14]. 

The ssvRNA SARS-CoV-2 virus possesses an enveloped, positive-stranded ssvRNA genome of about ~29,811 nt, and successful infection by SARS-CoV-2 initially causes severe and acute respiratory distress and highly lethal pneumonia known a COVID-19 (GenBank accession no. NC_045512.2) [15,16,17,18,19]. COVID-19 infection is often accompanied by multiple parallel or delayed systemic complications [17,19,20,21]. The SARS-CoV-2 RNA viral sequence is highly homologous to the ssvRNAs of severe acute respiratory syndrome coronavirus-1 (SARS-CoV-1) and Middle Eastern respiratory syndrome (MERS-CoV) which also cause acute respiratory pathology and multiple cardiovascular, neurological, and post-infection complications [17,18,19,20,21]. The SARS-CoV-2 virus is considerably larger than the size of the average cellular messenger RNA (mRNA; ~2000–5000 nt), but like single-stranded mRNA, appears to be intrinsically susceptible to complementary base-pair interaction with other ssRNAs, including sncRNA and miRNA (this interaction also involves RNA binding ribonucleoproteins and Argonaute (AGO) proteins, which guide the repression of mRNA targets [17,22,23,24,25,26,27].). For example, the first report for the affinity of any miRNA for the SARS-CoV-2 RNA genome was for the 23 nt human miRNA hsa-miRNA-5197-5p (encoded at chr 5q31.3; 5′-CAAUGGCACAAACUCAUUCUUGA-3′; www.mirbase.org/cgi-bin/mirna_entry.pl?acc=MIMAT0021130 accessed on 15 June 2021) that is over 90% homologous to a 3′ down-stream region of the SARS-CoV-2 RNA sequence [15,16,24,25,26]. Subsequent RNA sequencing and other studies have shown that at least ~160, 18–22 nt naturally occurring human miRNAs have perfect complementarity in the miRNA-mRNA ‘*seed region*’ to the single-stranded SARS-CoV-2 RNA genome; the top human host miRNA candidates targeting the SARS-CoV-2 ssvRNA genome include those of the miRNA-16, miRNA-21, miRNA-29a/b, let-7b, let-7e, miRNA-122, miRNA-146a microRNA families and others [17,24,25,26,27,28]. Interestingly, multiple studies have shown an elevation of the NF-kB-inducible, pro-inflammatory miRNA-146a in both Alzheimer’s disease (AD) and prion disease (PrD) and the selective induction of miRNA-146a by both human DNA and RNA neurotrophic viruses, including herpes simplex 1 (HSV-1; *Herpesviridae*; dsDNA genome), Hantavirus (HTV; *Bunyaviridae;* (−) ssRNA genome) and human immunodeficiency virus (HIV; *Retroviridae*; (+) ssRNA genome; [17,28,29,30,31]. Invasion of the human brain and CNS by ssvRNA and other neurotrophic viruses are associated with miRNA-mediated inflammatory neurodegeneration, disturbances in behavior, cognition, and dementia [29,30]. Whether viral invasion inducing miRNA-146a after infection is a protective mechanism of the human host cell, and/or if microRNAs, such as miRNA-146a, can target and inactivate ssvRNAs, such as SARS-CoV-2, is currently not well understood [28,29,30]. Exosomes carrying complex cargos of miRNA and/or interfering RNA that normally shuttle between various cell types also have a high potential to target specific sequence motifs of SARS-CoV-2 [31,32,33,34,35,36]. 

Overall these findings: **(i)** Provide insight into miRNA-mediated natural immunity via a prospective SARS-CoV-2 sense-miRNA-antisense interaction and host sncRNA-mediated regulatory mechanisms that are highly selective and protective against viral pathogens possessing an ssRNA genome; and **(ii)** illustrates the potential and novel use of host miRNAs or engineered, stabilized miRNAs as protective agents against SARS-CoV-2 invasion that may be useful in blocking ssvRNA viral replication in humans, and/or serve as sncRNA-based therapeutics against or biomarkers for SARS-CoV-2 infection [31,32,33,34,35,36,37,38,39]. 

## 2. Conclusions

A type of sncRNA sometimes referred to as interfering RNA (iRNA) has long been known to have evolved in plants, invertebrates, and vertebrates as an antiviral mechanism, combating viral infections through the rapid degradation of invading ssvRNA by binding to viral nucleic acid sequences to inhibit viral replication and host cell infection [33,35,36,37,38,39]. A human host’s natural and unique complement of miRNAs, sncRNAs, mRNAs, and other small RNA and/or DNA sequences appears to contribute to the observed natural quantity, complexity, and variability of nucleic acids located within host cells, and hence, their individual susceptibility to viral infection [11,12,13,14,32]. More specifically, the targeting and inactivation of SARS-CoV-2 by natural and/or pre-existing miRNAs or sncRNAs: **(i)** Would provide a natural protective mechanism or ssRNA-based innate-immune type of ‘*host immunity*’ against SARS-CoV-2 invasion, replication and COVID-19 [36,37]; **(ii)** would help explain individual variability of innate-immunity and resistance to SARS-CoV-2 infection of some persons who naturally express sncRNAs or miRNAs antisense to SARS-CoV-2, or other miRNAs or ssRNAs that target pathological ssRNA viruses [36,37,38,39,40]; **(iii)** suggests that very specific artificially synthesized and chemically stabilized sncRNA and/or miRNAs may be of therapeutic value in the accelerated destabilization, depolymerization, and destruction of the invading SARS-CoV-2 viral RNA sequence itself, resulting in SARS-CoV-2 degradation and the rapid cessation and termination of SARS-CoV-2 pathogenic activity; **(iv)** may also be useful in the targeting and inactivation of other contagious and invasive viruses, including neurotrophic viruses, that possess ssRNA genomes; and **(v)** would present a series of useful biomarkers to assess the absence or specific stage of SARS-CoV-2 infection and the efficacy of anti-SARS-CoV-2 pharmaceutical intervention and treatment strategies [31,39].

## 3. Summary

In summary, miRNAs have the potential to shape the host’s innate-immune response to SARS-CoV-2 infection. The predominant role of mammalian miRNAs is to decrease target mRNA levels, and the ssvRNA SARS-CoV-2, like mRNA, is likely to be targeted for degradation or modulation by a similar miRNA-mediated mechanism [1,23,24,25,26,27,28]. Because the abundance, speciation, and complexity of all miRNAs are both highly variable and unique to an individual, the immunological and physiological response to SARS-CoV-2 infection may also be unique to that individual, and form part of a sncRNA-mediated innate-immune defense. This could very well explain the significant variability in the individual response to SARS-CoV-2 invasion, the widely observed variation in the clinical presentation of COVID-19, and the extent of SARS-CoV-2-induced cellular and systemic pathology in the human host. 

Significance of the Research: Human genetic, epigenetic, and microbiome genome variability are an intrinsic property of each *Homo sapien* [7,10,11,12,13,14,35,39,40,41,42,43,44]. This individual diversity in genomic expression is reflected in part by the substantial heterogeneity of miRNA species abundance, sequence variety, and complexity in human cells, tissues, and physiological systems [11,12,13,14,35,36,39]. These single-stranded RNAs (ssRNAs) represent a ubiquitous form of sncRNAs found in all eukaryotic cells and tissues; their major function is to bind with single-stranded messenger RNA (mRNA) targets to form a transient miRNA-mRNA duplex which is rapidly degraded by ribonucleoproteins and nucleases within the cell, thus preventing productive translation at the eukaryotic 80S ribosome [1,2,3,4,5,7]. Therefore, mammalian miRNAs predominantly function to decrease target mRNA (ssRNA) levels, although several other miRNA- and Argonaute-2 (AGO2)-mediated gene regulatory mechanisms have been described [22,23,45].

Thus, the major functions of host miRNAs are: **(i)** In mRNA silencing; **(ii)** in the repression of the expression of genetic information encoded in the target mRNA of that miRNA; **(iii)** in the post-transcriptional regulation of gene expression, and this extends into shaping the transcriptome of the cell in both health and disease; and **(iv)** in playing a modulatory role in host interactions with viruses, including coronaviruses [6,13,34,44,46,47,48]. Because ssvRNA like the SARS-CoV-2 virus is also susceptible to recognition by miRNA, naturally-occurring miRNAs may function to seek out ssvRNA and neutralize their actions, thus contributing to a novel miRNA-facilitated innate-immunity in the host. Because different human individuals each have a heterogeneous repertoire of different miRNAs, it follows that each individual would be variably susceptible to the infectious pathobiology of various ssvRNA viruses, such as SARS-CoV-2. This may explain in part the significant, remarkable, and widely observed individual variability in sensitivity to SARS-CoV-2 and COVID-19, and each individual’s unique response to SARS-CoV-2 infection and susceptibility to or recovery from the invasion of that highly lethal viral pathogen.

## 4. Simple Summary

This commentary addresses an important and often overlooked immunological aspect and potentially unrecognized function of microRNAs (miRNAs) that have the potential to regulate infection by single-stranded viral RNA (ssvRNA), including the severe acute respiratory syndrome coronavirus-2 (SARS-Cov-2). Small non-coding RNAs (sncRNAs) like miRNAs are known to ‘*seek out*’ their single-stranded messenger RNA (mRNA) targets by base pair complementarity, typically by forming a transient double-stranded RNA followed by de-capping and de-adenylation of the target mRNA. With the cooperation of ribonucleoproteins, such as Argonaute 2 (AGO2) and the RNA-induced silencing complex (RISC), this eventually leads to destabilization and/or endonucleolytic degradation of that target mRNA and translational repression. Hence, the 2650 different miRNAs in human cells, besides serving as important post-transcriptional regulators of cellular gene expression, may also act as part of a modulatory ‘innate-immune’ mechanism against single-stranded RNA viruses, such as SARS-CoV-2, and perhaps other ssvRNA pathogens. Because miRNA abundance, speciation, and complexity varies widely among human individuals, this finding: **(i)** may explain in part the significantly variable immunological and pathological response of different humans to the ravages of SARS-CoV-2 infection; and **(ii)** contribute to the concept of ‘*human biochemical and genetic individuality*’, and the widely observed variable resistance of different individuals to highly infectious virally-transmitted disease mediated by ssvRNA.
